# Diagnosis–Treatment Discordance in Suspected Lyme Neuroborreliosis: A Retrospective Cohort Study from a Romanian Tertiary Centre (2008–2025)

**DOI:** 10.3390/pathogens15101072

**Published:** 2026-10-09

**Authors:** Cristina Alexandra Cheran, Laura Georgiana Tirlescu, Andreea Madalina Panciu, Adriana Hristea

**Affiliations:** 1 Faculty of Medicine, Carol Davila University of Medicine and Pharmacy, 020021 Bucharest, Romania; laura-georgiana.tirlescu@drd.umfcd.ro (L.G.T.); andreea-madalina.firtat@drd.umfcd.ro (A.M.P.); adriana.hristea@umfcd.ro (A.H.); 2 Department of Infectious Diseases, Bucharest Emergency University Hospital, 050098 Bucharest, Romania; 3 “Prof. Dr. Matei Bals” National Institute of Infectious Diseases, 021105 Bucharest, Romania

**Keywords:** antibiotic stewardship, *Borrelia burgdorferi*, diagnosis–treatment discordance, diagnostic criteria, Lyme neuroborreliosis

## Abstract

**Background**: European and Romanian recommendations for Lyme neuroborreliosis (LNB) emphasise cerebrospinal fluid (CSF) pleocytosis and a positive intrathecal antibody index (IAI) for diagnostic confirmation. We assessed whether antibiotic treatment in suspected LNB aligns with diagnostic recommendations. **Methods**: We conducted a retrospective, single-centre cohort study of patients with suspected LNB between 2008 and 2025, all with positive first-tier ELISA (enzyme-linked immunosorbent assay) *Borrelia* serology. Patients were classified as confirmed, probable, possible, or ruled-out LNB based on clinical presentation, serology, and CSF findings. We assessed factors associated with antibiotic treatment and temporal changes in diagnostic work-up and treatment patterns. **Results**: Among 58 patients, LNB was confirmed in 4 (6.9%), probable in 11 (19.0%), possible in 31 (53.4%), and ruled out in 12 (20.7%). Antibiotics were administered to 47 patients (81.0%), of whom 68.1% had possible or ruled out LNB. Antibiotic treatment was more frequent in early-stage than in late-stage disease (38/42, 90.5% vs. 9/16, 56.2%; *p* = 0.006); no significant association was detected with confirmed (*p* = 1.000) or ruled-out (*p* = 0.681) status. Between 2008 and 2019 and 2023–2025, IAI testing increased significantly (35.1% vs. 71.4%, *p* = 0.013), yet overall antibiotic use remained unchanged (81.1% vs. 81.0%, *p* = 1.000). **Conclusions**: Antibiotic treatment was poorly aligned with diagnostic classification, with most treated patients lacking confirmation. Improved diagnostic evaluation over time was not accompanied by a reduction in antibiotic use, highlighting a critical need for stronger diagnostic and antibiotic stewardship.

## 1. Introduction

Lyme neuroborreliosis (LNB) represents a neurological manifestation of *Borrelia burgdorferi* sensu lato infection, with a broad and often nonspecific clinical spectrum, which makes its diagnosis challenging in clinical practice. Clinical manifestations are heterogeneous, and seropositivity does not establish active neuroborreliosis.

With an increasing incidence of Lyme disease worldwide, influenced by ecological and climate changes affecting the vector populations, especially *Ixodes ricinus* in Europe, which is also the predominant tick species in Romania [1,2], the clinical management of suspected LNB cases has become increasingly complex.

Neurological involvement in LNB can manifest in two distinct temporal phases: early-onset (almost 90% of the cases) and late-onset. In the early phase, defined as a duration of symptoms of less than six months, manifestations include meningo-radiculo-neuritis, also known as Bannwarth syndrome, and involvement of the VII cranial nerve (less frequently, the III, IV, and VI cranial nerves), as well as encephalitis, myelitis, cerebral vasculitis, and peripheral neuritis [3].

In the late, or “chronic” phase, with a duration of symptoms longer than six months to years, patients can present with chronic meningitis, encephalomyelitis, radiculomyelitis, or late-onset neuropathy (mono-, radiculo-, or polyradiculopathy) often associated with sclerotic lesions such as acrodermatitis chronica atrophicans [4,5].

The diagnosis of LNB relies on specific case definitions. The EFNS (European Federation of Neurological Societies) 2009 criteria define **definite neuroborreliosis** by three criteria: neurological symptoms suggestive of LNB without other obvious reasons, cerebrospinal fluid (CSF) pleocytosis, and intrathecal *B. burgdorferi* antibody production, and **possible neuroborreliosis**, when only two of the three criteria are met [6].

The Romanian national surveillance methodology [7] maps closely onto the ESGBOR (the ESCMID Study Group for Lyme Borreliosis) recommendations for confirmatory diagnosis (the requirement of CSF pleocytosis together with intrathecal *Borrelia*-specific antibody synthesis) [8]. Under this methodology, the **confirmed** case requires CSF pleocytosis plus intrathecal antibody production (or direct detection in CSF); the **probable** case corresponds to CSF pleocytosis with serum and/or CSF antibodies but without demonstrated intrathecal synthesis, or intrathecal synthesis alone, and is flagged for retesting on paired serum/CSF samples after three to six weeks. The methodology does not include a serology-only “possible” tier, which we therefore defined specifically for this study; it also adds a paediatric rule (in children < 18 years, facial palsy or cranial neuritis with erythema migrans in the past two months and serum *Borrelia*-specific IgG is sufficient).

The diagnosis of LNB presents significant challenges. While two-tier serological testing (ELISA [enzyme-linked immunosorbent assay]/IFI [indirect immunofluorescence assay] followed by Western blot [WB]) is highly sensitive in true LNB (positive results in 97–100% of cases), its lack of specificity necessitates further investigation. Antibodies persist for years even after effective treatment or past infection, and IgM reactivity is prone to cross-reaction, producing false positives [9,10,11,12]. For this reason, ESGBOR complementary guidance further emphasises the rational use of laboratory testing in suspected LNB and requires CSF examination with intrathecal *Borrelia*-specific antibody synthesis (specific CSF/serum antibody index) alongside pleocytosis, while noting that the antibody index may still be negative in very early disease [8].

Therefore, CSF examination is a central component of diagnostic confirmation of LNB, particularly when distinguishing probable or confirmed disease from serology-supported clinical suspicion. PCR or culture of the CSF has low sensitivity, so a negative test cannot rule out the pathology [13,14]. The intrathecal antibody index (IAI), calculated as the ratio of specific IgG in CSF to specific IgG in serum, divided by the ratio of total IgG in CSF to total IgG in serum, reflects intrathecal production of specific antibodies. Its sensitivity is ~80% within the first six to eight weeks and approaches 100% thereafter, but, similar to serology, it can remain positive after effective treatment and is not a reliable marker of active disease [8,10].

According to the most recent national surveillance analysis, 1091 reportable Lyme borreliosis cases were registered in 2025 in Romania, 70% higher than the pre-pandemic five-year average (643 cases; 2017–2024, excluding the pandemic years) [15]. Most cases presented as early-stage, localised Lyme borreliosis, with only a small number of probable LNB cases reported, affecting both children and adults with manifestations such as facial nerve paralysis, lymphocytic meningitis, and radiculoneuritis.

Notably, despite the mandated reporting of Lyme disease to local Public Health Departments within five days after diagnosis (as recommended by the surveillance methodology), Lyme disease is likely significantly underreported in Romania [16,17].

Beyond under-reporting, the management of suspected LNB raises another distinct concern. Because most patients present with non-specific neurological complaints and only serological support, antibiotics are frequently prescribed under diagnostic uncertainty. Unnecessary courses, often administered intravenously, might expose patients to adverse events and contribute to antimicrobial resistance without benefit, which is why diagnostic and antibiotic stewardship is extremely important in the management of presumed Lyme disease cases [18,19]. There are limited data on whether treatment follows diagnostic certainty in Romanian tertiary care practice.

We therefore aimed to investigate whether antibiotic prescribing for suspected LNB aligns with objective diagnostic evidence. We stratified our cohort into four levels of diagnostic certainty (confirmed, probable, possible, or ruled-out). The confirmed, probable, and ruled-out categories followed the Romanian national surveillance methodology, whereas the “possible” category was defined specifically for the present study to accommodate patients with an incomplete CSF work-up; we then compared treatment approaches across these groups. As a secondary objective, we compared two time periods (2008–2019 vs. 2023–2025) to determine whether improvements in diagnostic work-up over time translated into more selective antibiotic use. Ultimately, by highlighting the gap between clinical suspicion and laboratory-confirmed neuroinflammation, we seek to promote a more evidence-based approach to diagnostic and antibiotic stewardship in suspected LNB.

## 2. Materials and Methods

### 2.1. Study Design and Setting

We conducted a retrospective, observational, single-centre cohort study at the National Institute for Infectious Diseases “Prof. Dr. Matei Balș”, Bucharest, Romania. Data were retrospectively extracted from the electronic medical records of patients admitted between 2008 and 2025.

Patients were eligible if they presented with clinical suspicion of LNB, had at least one compatible neurological manifestation prompting evaluation for Lyme borreliosis, and had sufficient data to assess diagnostic classification and treatment decisions. The minimum dataset included a documented neurological presentation, serological results, and a recorded antibiotic treatment decision. Both adults and children (<18 years) were included. In children, the national paediatric diagnostic rule was applied, requiring either facial palsy or cranial neuritis in the context of a recent history of erythema migrans.

All patients underwent two-tier serological testing. Only patients with a positive first-tier ELISA were included in the analytical cohort, with seropositivity defined as IgG reactivity (positive or borderline), with or without IgM reactivity. Isolated IgM positivity was not considered sufficient for inclusion. Patients were excluded if diagnostic or treatment data were insufficient, or confirmatory second-tier WB results were unavailable. Lumbar puncture (LP) was not required for study inclusion, allowing patients with incomplete CSF evaluation to be retained in the cohort and classified in the study-defined possible LNB category. Borderline first-tier ELISA IgG results were treated as reactive and, like positive results, proceeded to second-tier immunoblot confirmation; throughout the manuscript, “positive serology” refers consistently to a positive two-tier result (a positive or borderline ELISA confirmed by WB).

Because inclusion required inpatient evaluation with second-tier serological confirmation, the analysed cohort represents a selected population assessed at a single tertiary referral centre and is not intended to be representative of all patients with suspected LNB in Romania; the exclusion of outpatient-only patients and of patients without an available WB result may introduce selection and verification bias (addressed in Section 4).

### 2.2. Clinical Variables and Laboratory Evaluation

Demographic and epidemiological variables included age, sex, history of tick exposure, and documented history of erythema migrans. Disease stage was classified as **early LNB** when neurological manifestations occurred within the early disseminated phase of infection and as **late LNB** when manifestations were compatible with late disease (symptoms duration > 6 months).

Clinical manifestations were recorded according to the Romanian National Methodology for Lyme disease surveillance and included meningitis, encephalomyelitis, facial palsy, optic neuritis or other cranial neuritis, and radiculoneuropathies, including Bannwarth syndrome, and peripheral neuropathy.

Serological and cerebrospinal fluid (CSF) findings were used to establish the diagnostic certainty category and were also recorded as study variables for descriptive and comparative analyses. When LP was performed, CSF was assessed for total leukocyte count and differential cell count, protein and albumin concentrations, glucose concentration, and the CSF/serum glucose ratio based on simultaneous serum glucose measurement. CSF pleocytosis and blood–CSF barrier dysfunction were assessed according to the laboratory reference ranges.

Intrathecal synthesis of *B. burgdorferi* sensu lato-specific antibodies was assessed using an antibody index determined in an external laboratory by quantitative measurement of *Borrelia*-specific IgG and IgM antibodies in paired CSF and serum samples. An antibody index > 1.5 was considered positive.

Neuroimaging findings, including computed tomography and magnetic resonance imaging, were recorded when available. Treatment data included antibiotic regimen, dose, duration of therapy, and concomitant corticosteroid use.

### 2.3. Case Definitions and Diagnostic Classification

Final diagnostic classification was based on the Romanian national surveillance methodology [7]. Because this methodology does not include a serology-only category, we additionally defined the “possible LNB” category specifically for the present study (Table 1).

**Confirmed LNB** was defined as compatible neurological manifestations and positive *Borrelia*-specific serum antibodies based on two-tier testing plus evidence of an inflammatory CSF syndrome, characterised by lymphocytic pleocytosis and/or blood–CSF barrier dysfunction, together with evidence of intrathecal *Borrelia*-specific antibody synthesis (antibody index > 1.5) or direct detection of *Borrelia* by PCR or culture in CSF.

**Probable LNB** was defined as compatible neurological manifestations and positive *Borrelia*-specific serum antibodies based on two-tier testing plus either evidence of an inflammatory CSF syndrome, characterised by lymphocytic pleocytosis and/or blood–CSF barrier dysfunction, or demonstrated intrathecal *Borrelia*-specific antibody synthesis.

**Possible LNB** was defined as compatible neurological manifestations and positive *Borrelia*-specific serum antibodies in patients who did not fulfil the criteria for probable or confirmed LNB because CSF evaluation was unavailable, incomplete, or did not demonstrate the required diagnostic findings (no LP, LP with no pleocytosis, or LP with incomplete testing).

These criteria correspond to the confirmatory requirements of the ESGBOR laboratory recommendations and the EFNS “definite” category. CSF inflammation (lymphocytic pleocytosis and/or blood–CSF barrier dysfunction) and intrathecal *Borrelia*-specific antibody synthesis were treated as two separate criteria, with confirmed LNB requiring both and probable LNB requiring either one. In this cohort, no patient was classified as probable or confirmed on the basis of blood–CSF barrier dysfunction (an isolated elevated albumin quotient) alone: 14 of the 15 probable/confirmed patients had lymphocytic pleocytosis, and the single exception was classified on the basis of positive IAI together with a positive WB. An isolated elevation of the albumin quotient, in the absence of pleocytosis, was therefore not used as the sole marker of neuroinflammation.

Patients were classified as **ruled out** when confirmatory (second-tier) WB was negative and/or an alternative diagnosis adequately explaining the neurological presentation was established.

### 2.4. Antibiotic Treatment

Antibiotic treatment was considered the primary management outcome. Treatment decisions were assessed according to final diagnostic classification, disease stage, and available laboratory findings. The proportion of patients receiving antibiotics was compared across diagnostic categories and clinical subgroups. Because antibiotic treatment was frequently initiated before completion of the diagnostic work-up, diagnosis–treatment concordance was assessed retrospectively against the final diagnostic classification rather than as a direct assessment of real-time prescribing decisions.

To describe the relationship between antibiotic prescribing and diagnostic certainty, every treated patient was assigned to one of three mutually exclusive categories of diagnosis–treatment concordance, based on the final diagnostic classification together with the recorded regimen and treatment duration. “Concordant antibiotic treatment” was defined as antibiotic therapy administered to a patient with confirmed or probable LNB using a guideline-concordant regimen and duration ≤ 28 days. “Treatment under diagnostic uncertainty” was defined as antibiotic treatment given to a patient in the possible LNB category, in whom neuroborreliosis could neither be confirmed nor excluded on the basis of the available serological and CSF findings, so that the indication for treatment remained uncertain. “Discordant antibiotic treatment” was defined as treatment that was discordant with the final retrospective diagnostic classification or delivered as a regimen discordant with guideline-recommended therapy, comprising any of the following: (i) treatment of a patient in whom LNB had been ruled out and an alternative diagnosis not requiring antibiotics had been established; (ii) an excessive treatment duration (>28 days); or (iii) an unjustified combination of ceftriaxone and doxycycline. Patients who did not receive antibiotics were not classified. These categories describe the concordance between the treatment decision and the final diagnostic classification and should not be interpreted as a direct measure of clinically (in)appropriate treatment. The unit of analysis for treatment concordance was the individual patient: each treated patient was assigned to a single category on the basis of the entire antibiotic treatment received (including any second course), rather than analysing individual courses separately.

### 2.5. Statistical Analysis

Statistical analyses were performed using IBM SPSS (Statistical Product and Service Solutions) Statistics, version 26 (IBM Corp., Armonk, NY, USA). Missing values, coded as 999 in the original dataset, were treated as missing data, and each variable was analysed using complete cases.

The distribution of continuous variables was assessed using the Shapiro–Wilk test. Normally distributed variables (age) were summarised as mean ± standard deviation and compared with one-way ANOVA, whereas non-normally distributed variables (length of stay) were summarised as median and interquartile range (IQR) and compared with the Kruskal–Wallis test.

Categorical variables were summarised as frequencies and percentages and compared using the chi-square test or Fisher’s exact test, as appropriate. Fisher’s exact test was used when expected cell counts were <5, and the Fisher–Freeman–Halton exact test was used for comparisons across the three diagnostic groups.

Temporal comparisons between the two periods used Fisher’s exact test, with associations expressed as odds ratios and 95% confidence intervals for the 2023–2025 period relative to 2008–2019.

All statistical tests were two-sided, and *p* < 0.05 was considered statistically significant. Analyses were considered exploratory and hypothesis-generating. No formal a priori sample-size calculation was performed, as is appropriate for a retrospective descriptive cohort. Exact tests were used throughout because of small expected cell counts.

## 3. Results

### 3.1. Cohort Characteristics and Clinical Presentation

**Figure 1:**
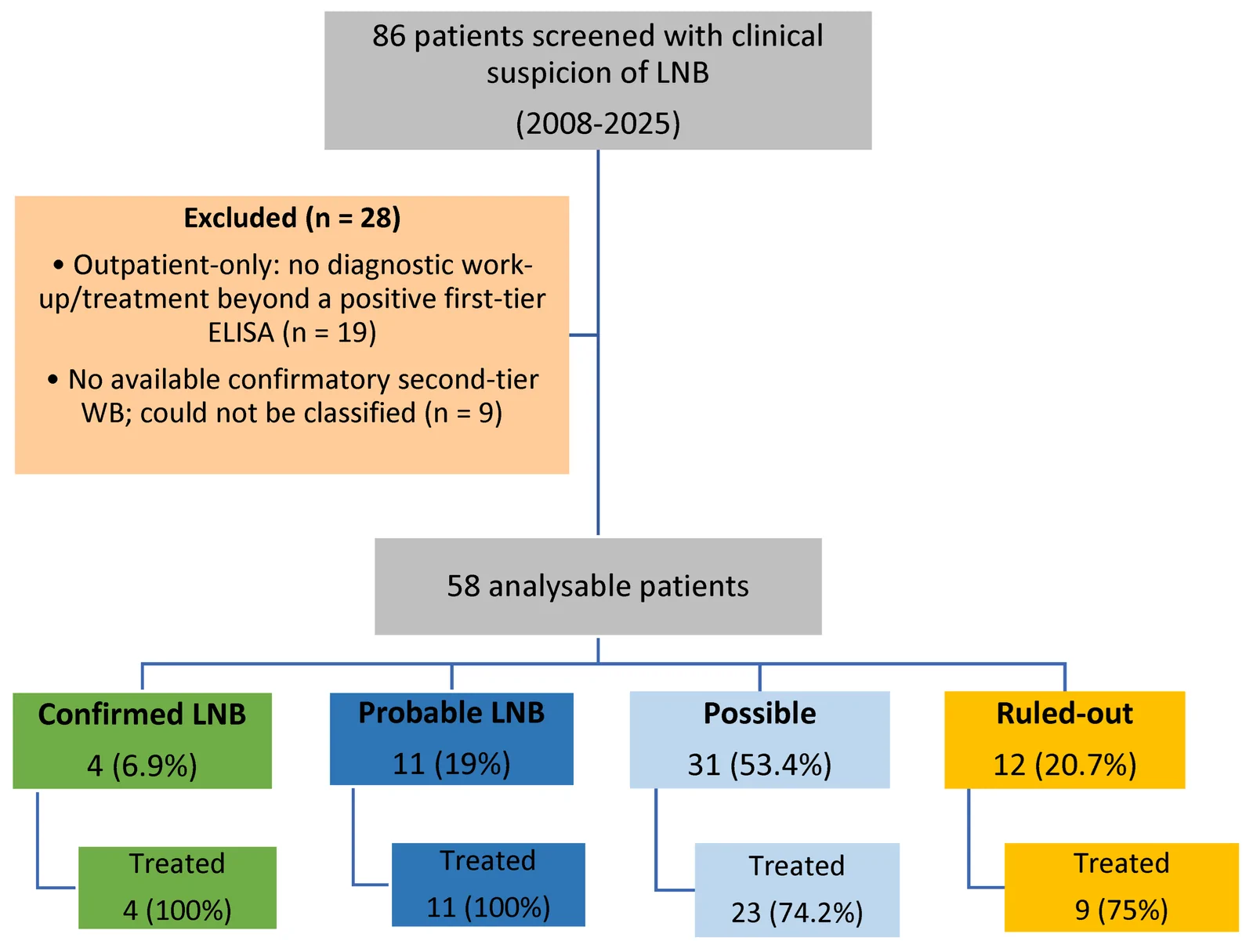
Patient selection and diagnostic classification (STROBE flow).

Of 86 patients evaluated for clinical suspicion of LNB, 19 (22.1%) were managed exclusively in the outpatient setting, with no diagnostic work-up or treatment recorded beyond a positive first-tier ELISA, and were therefore excluded. A positive first-tier ELISA was the inclusion criterion; however, nine further patients were excluded because confirmatory second-tier WB testing, required to establish the final diagnostic classification, was not available. The remaining 58 patients constituted the analytical cohort (Figure 1).

The mean age was 40.3 ± 15.3 years (median 38 years, range (min–max) 7–68 years), and 32 (55.2%) were female. Two (3.4%) were paediatric patients. Median hospital stay was 18 days (IQR, 6–26). Thirty-seven patients were admitted during 2008–2019 and 21 during 2023–2025. No eligible cases were identified in 2018 or during 2020–2022. The number of patients per year was four (2008), three (2009), one (2010), seven (2011), six (2012), five (2013), three (2014), three (2017) and five (2019) in the first period, and four (2023), nine (2024) and eight (2025) in the second; no eligible cases occurred in 2015, 2016, 2018, or 2020–2022.

A history of tick bites and erythema migrans was documented in 11 patients (19.0%). Overall, 42 patients (72.4%) were classified as having early-stage disease according to the predefined study criteria. Of the 16 (27.6%) patients presenting with a clinical picture suggestive of late-stage disease (symptoms persistent for more than six months), 11 (68.8%) were categorised as possible and 4 (25%) were finally ruled out. Clinical and demographic characteristics according to diagnostic classification are presented in Table 2.

Radiculoneuropathy was the most frequent neurological manifestation, occurring in 52 patients (89.7%). Facial palsy was documented in 7 (12.1%), optic neuritis in 13 (22.4%), meningitis in 6 (10.3%), and encephalomyelitis in 2 (3.4%). Radiculoneuropathy was also frequent among patients ultimately classified as ruled-out LNB (11/12, 91.7%), indicating its limited specificity when considered in isolation.

### 3.2. Diagnostic Work-Up and Diagnostic Classification

Lumbar puncture was performed in 50 of the 58 patients (86.2%). CSF pleocytosis was identified in 16 patients (32%) and hyperproteinorrachia in 19 (38%). Pleocytosis was substantially more frequent among patients classified as probable/confirmed LNB than among those with possible or ruled-out disease (93.3% vs. 0% and 22.2%, respectively; Table 1).

IAI *Borrelia*-specific testing was performed in 28 patients (48.3%) and was positive in 5. All positive IAI results occurred among patients classified as probable or confirmed LNB. All patients underwent two-tier serological testing (ELISA followed by confirmatory WB). A positive two-tier result (a positive or borderline ELISA confirmed by a positive WB) defined the seropositive categories (confirmed, probable, and possible LNB), whereas ELISA-positive patients with a negative confirmatory WB were classified as ruled out, and patients in whom no WB result was available were excluded.

Overall, 4 patients (6.9%) met the criteria for confirmed LNB, 11 (19%) for probable LNB, 31 (53.4%) for possible LNB, and 12 (20.7%) were classified as ruled out. Thus, only 15/58 patients (25.9%) fulfilled probable or confirmed diagnostic criteria.

The possible LNB category (*n* = 31) was clinically heterogeneous with respect to the completeness of CSF evaluation: 5 patients (16.1%) had no LP, 10 (32.3%) underwent LP, had no pleocytosis, and had no IAI assessment, and 16 (51.6%) underwent LP, had no pleocytosis, and had an IAI requested but not positive (the result being externalised/unavailable in 14 and negative in 2). By definition, none of the possible-LNB patients had documented CSF pleocytosis. This category was therefore populated largely by patients with an incomplete rather than a negative diagnostic work-up.

Among patients classified as probable LNB, 10/11 had CSF pleocytosis, the single exception being a patient with late-stage disease who instead demonstrated a positive antibody index and a positive WB. All four confirmed cases had CSF pleocytosis and evidence of intrathecal antibody synthesis. In contrast, CSF inflammation was uncommon among patients classified as possible LNB.

Neuroimaging was performed in 28 patients (48.3%). CT was performed in 10 patients, with abnormalities reported in 2, while MRI was performed in 22 patients, with abnormalities reported in 14. Most imaging abnormalities were non-specific.

### 3.3. Antibiotic Treatment and Diagnostic-Treatment Concordance

Despite only 4 patients (6.9%) having confirmed LNB, 47/58 (81%) received antibiotic treatment. Among treated patients, 32/47 (68.1%) had either possible or ruled-out LNB, including 23 patients with possible LNB and 9 with ruled-out disease. Overall, 43/47 treated patients (91.5%) did not have confirmed LNB.

Antibiotic treatment was not significantly associated with diagnostic certainty. Treatment was administered to all patients with probable/confirmed LNB, 23/31 (74.2%) with possible LNB, and 9/12 (75.0%) with ruled-out LNB (*p* = 0.063). Similarly, treatment was not significantly associated with either confirmed LNB (*p* = 1.000) or ruled-out LNB (*p* = 0.681).

Treatment was instead associated with clinical presentation. Antibiotics were administered to 38/42 (90.5%) patients with early-stage disease, compared with 9/16 (56.2%) with late-stage disease (*p* = 0.006).

Ceftriaxone was the first-line agent in 37 treated patients and doxycycline in 10 (47 patients in total). Ceftriaxone was given as monotherapy in 25 patients and doxycycline as monotherapy in 8 patients, while 14 patients received both ceftriaxone and doxycycline (12 sequentially and 2 in combination). The median duration of antibiotic treatment was 21 days (IQR, 10–28). Nine patients (15.5%) also received corticosteroids. Overall, 14 patients (24.1%) received a second antibiotic course (6 with possible, 5 with probable, 1 with confirmed LNB, and 2 with ruled-out LNB).

### 3.4. Temporal Changes in Diagnostic Evaluation and Treatment

The diagnostic work-up improved during the study period. The proportion of patients undergoing intrathecal antibody index testing increased from 35.1% in 2008–2019 to 71.4% in 2023–2025 (*p* = 0.013), while LP rates remained similar (83.8% vs. 90.5%; *p* = 0.698).

Despite the increased use of intrathecal antibody testing, the proportion of patients meeting the criteria for confirmed LNB did not change significantly between periods (5.4% vs. 9.5%; *p* = 0.615). Similarly, antibiotic use remained high and did not decrease significantly, occurring in 30/37 patients (81.1%) during 2008–2019 and 17/21 (81.0%) during 2023–2025 (*p* = 1.000). Thus, although the diagnostic evaluation became more comprehensive over time, this improvement was not accompanied by a significant reduction in antibiotic prescribing (Table 3).

Antibiotic prescribing frequently differed from diagnostic certainty (Table 4). Of the 47 treated patients, only 12 (25.5%) received treatment classified as “concordant”, all of them in the confirmed (three patients) or probable (nine patients) categories. Antibiotic use was classified as “treatment under diagnostic uncertainty” in 23 patients (48.9%), corresponding to the entire treated possible-LNB group, and as “discordant antibiotic treatment” in 12 (25.5%). The latter comprised nine patients with diagnosis ruled out, treated discordantly with the final diagnostic classification, together with three patients with confirmed or probable LNB, one who received an excessively long course (>28 days) and two who received an unjustified ceftriaxone–doxycycline combination. Overall, 35 of 47 treated patients (74.5%) were classified as “treatment under diagnostic uncertainty” or “discordant antibiotic treatment”. This proportion reflects the degree of concordance between the treatment decision and the final diagnostic classification rather than a direct measure of clinically unjustified treatment.

When treated patients were classified by diagnosis–treatment concordance (Table 4), the proportion judged concordant did not change significantly between periods (6/30, 20.0% in 2008–2019 vs. 6/17, 35.3% in 2023–2025; *p* = 0.306). Treatment under diagnostic uncertainty remained the largest category in both periods (13/30, 43.3% vs. 10/17, 58.8%; *p* = 0.371). Discordant antibiotic treatment, by contrast, declined significantly over time, from 11/30 (36.7%) of treated patients in 2008–2019 to 1/17 (5.9%) in 2023–2025 (*p* = 0.034), driven mainly by fewer treated patients in the ruled-out group (8 vs. 1).

## 4. Discussion

In this cohort, diagnostic classification followed the Romanian surveillance methodology (with a study-defined “possible” category), yet the treatment decision was largely independent of it. Antibiotics were prescribed to 47 of 58 patients (81.0%) while only 4 (6.9%) met confirmatory criteria. More than 90% of treated patients (43/47, 91.5%) received antibiotics without confirmation, including 9 of the 12 patients in whom LNB had been ruled out. We did not detect a statistically significant association between ruled-out or confirmed status and treatment (*p* = 0.681 and *p* = 1.000, respectively); given the near-universal prescribing (a ceiling effect) and the small numbers, these comparisons had limited statistical power and wide confidence intervals, so this should not be read as evidence of no association.

Although treatment did not follow diagnostic certainty, it tracked clinical alarm features. Antibiotic receipt was significantly associated with early-stage presentation (90.5% vs. 56.2%, *p* = 0.006). These findings are consistent with treatment decisions being influenced more strongly by clinical presentation than by the eventual diagnostic classification. Reassuringly, early-stage presentation, the feature that most drove treatment, is precisely one that is genuinely associated with confirmed LNB: in the largest Romanian cohort, meningitis/encephalitis/myelitis was significantly more frequent in definite/possible LNB, and shorter symptom duration likewise characterised confirmed disease [16,20].

This discordance appears to arise from a chain of weak links. The dominant presenting symptom, radiculoneuropathy, was the most frequent (89.7%) but also the least specific, occurring in most ruled-out patients and therefore unable to discriminate between different LNB categories. The CSF work-up required for confirmation was incompletely pursued, with about one in seven patients (8/58, 13.8%) having no LP performed and the IAI obtained in under half (48.3%).

Our confirmation rate aligns with the few comparable cohorts despite differing classification frameworks. In a Parisian referral centre, fewer than 10% of patients assessed for presumed Lyme borreliosis were confirmed, yet more than 80% of prescribed antibiotic courses proved unjustified [18]. Another French cohort of 155 patients from an endemic area confirmed definite/probable LNB cases in only 29% [20]. In the largest Romanian series, only 1.65% of suspected patients were classified as definite and 29% as definite/possible LNB, with LNB excluded in 71% according to EFNS criteria [16]. Our rate of definite LNB (6.9%) is of the same order, but our combined possible/probable group was larger (72.4%), reflecting the deliberate retention of patients admitted on clinical suspicion regardless of work-up completeness. A broad, serology-anchored “possible” category is precisely the reservoir in which the treatment decision detaches from confirmation.

At first glance, our finding that antibiotics were frequently prescribed to patients without confirmed LNB may appear to contradict the prevailing message that LNB is under-diagnosed and under-reported in Romania, where a single referral centre identifies more possible cases than are reported nationally, and the estimated under-detection multiplier for symptomatic *B. burgdorferi* sensu lato infection reaches 10.5 [16,17]. In fact, the two phenomena are complementary faces of a single problem; an incomplete confirmatory work-up. When the criterion-defining tests, CSF pleocytosis and the IAI, are not systematically obtained, true cases fail to be formally confirmed (thus increasing under-reporting), while clinically suspected but unconfirmed cases are treated anyway (increasing the treatment of unconfirmed disease); both are populated by the same over-inclusive “possible” category.

Intravenous ceftriaxone was the first-line agent in the large majority of treated patients (37 of 47). Two randomised trials have shown that oral doxycycline is non-inferior/equivalent to intravenous ceftriaxone for European LNB, with comparable clinical recovery and CSF normalisation [21,22]. Unnecessary antibiotic treatment is not harmless. Beyond selection for antimicrobial resistance, inappropriate courses (especially prolonged IV regimens) have caused *Clostridioides difficile* colitis, intravenous line-associated bloodstream infection, and, in severe cases, septic shock and death [19,23]. In our cohort, 43 of 47 treated patients (91.5%) received antibiotics without diagnostic confirmation. Intravenous ceftriaxone remains an appropriate option in selected clinical circumstances; its predominant use nonetheless represents a potential target for antimicrobial stewardship review, particularly regarding whether oral therapy could have been considered in individual patients when consistent with contemporary recommendations and their clinical status. Although these specific adverse events were not measured in our study, this pattern of near-universal empirical treatment for unconfirmed cases represents potentially avoidable antibiotic exposure, particularly among possible and ruled-out cases.

Although overall antibiotic prescribing remained unchanged, the pattern of prescribing changed: treatment of patients classified as ruled out decreased substantially over time. IAI testing roughly doubled, rising from 35.1% to 71.4% (*p* = 0.013), yet neither the confirmation rate (5.4% to 9.5%; *p* = 0.615) nor overall antibiotic use (81.1% to 81.0%; *p* = 1.000) changed. Notably, however, the diagnosis–treatment concordance profile did shift: discordant antibiotic treatment fell significantly, from 11/30 (36.7%) of treated patients in 2008–2019 to 1/17 (5.9%) in 2023–2025 (*p* = 0.034), driven mainly by fewer treated patients in the ruled-out group. Thus, better test ordering was accompanied by more restraint in clearly excluded cases, but not by an overall reduction in prescribing. Given the unequal duration of the two periods, the absence of eligible cases in several calendar years, and possible changes in referral case-mix, these temporal differences should be regarded as observed changes within this cohort rather than evidence of a causal change in diagnostic practice.

The external processing of the *Borrelia*-specific antibody index may have contributed to this pattern, as results were not necessarily available when treatment decisions were made. Turnaround times meant that these results were frequently unavailable at the point of the treatment decision, which plausibly explains why the antibody index was obtained in only 48.3% of patients and why clinicians defaulted to empirical treatment. This is a health system rather than a purely clinical driver of the diagnosis–treatment discordance: on-site or reflex antibody index testing of CSF, with results available before the prescribing decision, is an intervention that could convert improved test ordering into more selective treatment, the very translation that the temporal analysis showed was missing.

Several caveats temper the interpretation of the diagnosis–treatment concordance categories. Antibiotic treatment was frequently initiated before the diagnostic work-up was complete: CSF analysis and, in particular, the IAI (processed externally, with variable turnaround) were often unavailable at the point of the prescribing decision. Our categories are therefore based on the final, retrospective classification and describe the concordance between treatment and the eventual diagnosis, rather than the appropriateness of the real-time decision made under uncertainty. This caveat applies most to the “treatment under diagnostic uncertainty” category, which reflects treatment of patients in whom LNB could ultimately neither be confirmed nor excluded. By contrast, discordant antibiotic treatment defined by excessive duration (>28 days) or an unjustified ceftriaxone–doxycycline combination reflects the completed course, and treatment of ruled-out patients reflects treatment discordant with the final ruled-out classification; because the alternative diagnosis may, in some cases, have been established only during or after the antibiotic course, this category should be interpreted as discordance with the final diagnosis rather than as treatment knowingly continued against an already established diagnosis, while still flagging potentially avoidable antibiotic exposure. We therefore present “concordant antibiotic treatment”, “treatment under diagnostic uncertainty” and “discordant antibiotic treatment” as measures of diagnosis–treatment concordance, not as judgments of individual real-time clinical decisions.

Earlier Romanian work characterised the differential diagnostic spectrum and multidisciplinary management of these patients but did not capture the antibiotic treatment decision itself, as therapy was largely prescribed on an ambulatory basis [16,24]. Our study addresses a different question: whether the antibiotic treatment decision is aligned with the level of diagnostic certainty, and how diagnostic work-up and treatment patterns changed over time. To our knowledge, this specific question, treatment-confirmation concordance and its temporal evolution, has not previously been examined in a Romanian cohort. Set against near-universal prescribing, our data identify where in the pathway stewardship should intervene, since empiric or discordant, often intravenous courses carry adverse event and antimicrobial resistance costs without patient benefit [19].

A further limitation of our study is the small cohort of patients from a single center, limiting the power of association. Missing data were frequent, particularly for imaging, CSF parameters, and some WB results. Of 86 suspected patients, 19 (22.1%) evaluated exclusively as outpatients and 9 without second-tier WB confirmation could not be classified and were excluded. However, the inability to even classify a substantial fraction of suspected cases is itself a manifestation of the incomplete diagnostic documentation that our study describes. Our cohort also showed weaker epidemiological anchoring, with tick bites and erythema migrans being recalled in only 19%. It was further characterised by a relatively larger proportion of late-stage presentations, with 12 patients from the possible/probable group (20.7% of total participants) in contrast with classic descriptions in which up to 90% of LNB cases present in the early phase. Both observations are consistent with a tertiary referral population and with the low tick bite recall reported in other suspected-LNB cohorts [16]. Because treatment often preceded completion of the work-up, and the timing of individual test results relative to prescribing was not systematically retrievable, our findings describe diagnosis–treatment concordance and are descriptive and hypothesis-generating rather than a measure of real-time decision quality.

The study is therefore subject to potential selection and verification bias: excluding outpatient-only cases and patients without confirmatory WB may have enriched the cohort for more severe or diagnostically complex presentations and influenced the observed diagnostic and treatment patterns. In addition, the definition of discordant treatment is dependent on the final diagnostic classification, which introduces a degree of circularity, and retrospective data cannot capture the actual clinical reasoning or the factors underlying individual prescribing decisions. These findings should therefore be interpreted in light of the single-centre design, the relatively small sample size, and the large, heterogeneous “possible LNB” category. Given this heterogeneity and the incomplete availability of IAI results, findings concerning the possible LNB category should be interpreted with particular caution and not as findings from a homogeneous diagnostic group.

## 5. Conclusions

Among patients evaluated for suspected LNB at a single Romanian tertiary centre, antibiotic treatment was largely uncoupled from the diagnostic classification: prescribing was near-universal and concentrated in unconfirmed, serology-only presentations. Over the study period, a more complete diagnostic work-up was accompanied by a significant reduction in the treatment of clearly ruled-out cases, but not by an overall decline in antibiotic use, indicating that better testing alone did not translate into consistently more selective prescribing. Because treatment frequently preceded completion of the work-up, these findings describe diagnosis–treatment concordance rather than the appropriateness of individual real-time decisions. They nonetheless argue for standardised CSF-based confirmation, greater restraint in serology-only cases, and antibiotic stewardship (including timely, ideally on-site, antibody index testing and preferential oral therapy where appropriate) in the management of suspected LNB.

## Figures and Tables

**Table 1:** Case definitions and diagnostic classification.

Category	Clinical	Serology	CSF Inflammation	Intrathecal Antibodies/Direct Detection (IAI/DD)
Confirmed	+	+	+	+
Probable	+	+	(+ and IAI/DD −) or (− and IAI/DD +)	− or +
Possible *	+	+	CSF evaluation unavailable, incomplete, or did not demonstrate the required diagnostic findings	CSF evaluation unavailable, incomplete, or did not demonstrate the required diagnostic findings
Ruled out	+	+/−	+/− but alternative diagnosis	−/unavailable

* study-defined category; not part of the Romanian National case definition. Abbreviations: CSF, cerebrospinal fluid; IAI, intrathecal antibody index; DD, direct detection (PCR or culture); +, present; −, absent; +/−, present or absent.

**Table 2:** Cohort characteristics by diagnostic category.

Characteristic	Total (*n* = 58)	Probable/Confirmed (*n* = 15)	Possible (*n* = 31)	Ruled-Out (*n* = 12)	*p* ^1^
**Demographics**					
Age, years—mean ± SD	40.3 ± 15.3	48.3 ± 16.9	38.4 ± 14.4	35.3 ± 12.4	0.051
Female sex	32 (55.2%)	8 (53.3%)	19 (61.3%)	5 (41.7%)	0.500
**Clinical course**					
Length of stay, days—median (IQR)	18 (6–26)	21 (18–29)	18 (4–22)	8 (4–16)	0.036
Early-stage disease	42 (72.4%)	14 (93.3%)	20 (64.5%)	8 (66.7%)	0.096
**Neurological manifestations**					
Meningitis	6 (10.3%)	4 (26.7%)	0 (0%)	2 (16.7%)	0.005
Optic neuritis	13 (22.4%)	3 (20.0%)	7 (22.6%)	3 (25.0%)	1.000
Facial palsy	7 (12.1%)	3 (20.0%)	3 (9.7%)	1 (8.3%)	0.559
Radiculoneuropathy	52 (89.7%)	13 (86.7%)	28 (90.3%)	11 (91.7%)	1.000
Encephalomyelitis	2 (3.4%)	1 (6.7%)	1 (3.2%)	0 (0.0%)	1.000
**CSF findings**					
Lumbar puncture performed	50 (86.2%)	15 (100.0%)	26 (83.9%)	9 (75.0%)	0.116
CSF pleocytosis ^2^	16 (32.0%)	14 (93.3%)	0 (0.0%)	2 (22.2%)	<0.001
Hyperproteinorrachia ^2^	19 (38.0%)	10 (66.7%)	5 (19.2%)	4 (44.4%)	0.008
Antibody index performed	28 (48.3%)	8 (53.3%)	16 (51.6%)	4 (33.3%)	0.535
Positive antibody index ^3^	5 (17.9%)	5 (62.5%)	0 (0.0%)	0 (0.0%)	0.001
Antibiotic treatment	47 (81.0%)	15 (100%)	23 (74.2%)	9 (75.0%)	0.063
Second course of antibiotic	14 (24.1%)	6 (40.0%)	6 (19.4%)	2 (16.7%)	0.279
Combination antibiotic treatment	2 (3.4%)	2 (13.3%)	0 (0.0%)	0 (0.0%)	0.103
Corticosteroids	9 (15.5%)	3 (20.0%)	4 (12.9%)	2 (16.7%)	0.889

^1^ *p*-values: age—one-way ANOVA; length of stay—Kruskal–Wallis test; categorical variables, Fisher-Freeman-Halton exact test. ^2^ CSF findings (pleocytosis, hyperproteinorrachia) were compared among the 50 patients who underwent lumbar puncture; ^3^ the antibody index among the 28 patients tested. Abbreviations: SD, standard deviation; IQR, interquartile range; CSF, cerebrospinal fluid; LP, lumbar puncture; IAI, intrathecal antibody index. Treatment-related variables (antibiotic treatment, second antibiotic course, combination therapy, corticosteroids) are expressed relative to the whole cohort (*n* = 58) in the Total column and relative to each diagnostic subgroup in the subgroup columns. Bold indicates variable groupings (Demographics; Clinical course; Neurological manifestations; CSF findings).

**Table 3:** Comparison of diagnostic and treatment indicators between periods.

Indicator	2008–2019 (*n* = 37)	2023–2025 (*n* = 21)	*p*	OR
Lumbar puncture, *n* (%)	31 (83.8%)	19 (90.5%)	0.698	1.84 (0.34–10.06)
Antibody index tested, *n* (%)	13 (35.1%)	15 (71.4%)	0.013	4.62 (1.44–14.76)
Early-stage disease, *n* (%)	26 (70.3%)	16 (76.2%)	0.764	1.35 (0.40–4.62)
Antibiotic treatment, *n* (%)	30 (81.1%)	17 (81.0%)	1.000	0.99 (0.25–3.88)
Concordant antibiotic treatment *n* (%) *	6 (20%)	6 (35.3%)	0.306	2.18 (0.57–8.31)

LP = lumbar puncture. * = number/treated. Odds ratios (OR) are expressed for the 2023–2025 period relative to 2008–2019 (reference category). Only the increase in IAI testing was statistically significant (OR 4.62; 95% CI 1.44–14.76, excluding the null value); for all other indicators the 95% CIs were wide and crossed 1 (LP 1.84, 0.34–10.06; early-stage disease 1.35, 0.40–4.62; antibiotic treatment 0.99, 0.25–3.88; concordant treatment 2.18, 0.57–8.31), indicating imprecise, non-significant estimates.

**Table 4:** Diagnosis–treatment concordance of antibiotic treatment by diagnostic category.

Indicator	Confirmed (*n* = 4)	Probable (*n* = 11)	Possible (*n* = 31)	Ruled-Out (*n* = 12)	Total (*n* = 58)
Number of patients treated	4	11	23	9	47
Concordant antibiotic treatment	3	9	0	0	12
Treatment under diagnostic uncertainty	0	0	23	0	23
Discordant antibiotic treatment	1	2	0	9	12

## Data Availability

The original contributions presented in the study are included in the article, further inquiries can be directed to the corresponding author.

## Abbreviations

The following abbreviations are used in this manuscript:

ACA	Acrodermatitis chronica atrophicans
CNS	Central Nervous System
CSF	Cerebrospinal fluid
EFNS	European Federation of Neurological Societies
ELISA	Enzyme-linked immunosorbent assay
ESCMID	European Society of Clinical Microbiology and Infectious Diseases
IAI	Intrathecal antibodies index
IFI/IFA	Indirect Immunofluorescence Assay
IgM/G	Immunoglobulin M/G
IQR	Interquartile Range
LNB	Lyme neuroborreliosis
LP	Lumbar puncture
OR	Odds Ratio
SD	Standard Deviation
SPSS	Statistical Product and Service Solutions
WB	Western blot
